# Is Celiac Disease (CD) Prevalent in Patients with Multiple Sclerosis (MS): A Systematic Review and Meta-Analysis

**DOI:** 10.1155/2022/7091140

**Published:** 2022-12-10

**Authors:** Hamide Olfati, Hamed Ghoshouni, Narges Ebrahimi, Aida Mohammadi, Mahsa Ghajarzadeh

**Affiliations:** ^1^Department of Endocrinology, Razi Hospital, Qazvin, Iran; ^2^Faculty of Medicine, Shahid Sadoughi University of Medical Sciences, Yazd, Iran; ^3^Multiple Sclerosis Research Group (MSRG), Universal Scientific Education and Research Network (USERN), Tehran University of Medical Sciences, Tehran, Iran; ^4^Department of Neurology, Johns Hopkins University, Baltimore, MD, USA; ^5^Universal Council of Epidemiology (UCE), Universal Scientific Education and Research Network (USERN), Tehran University of Medical Sciences, Tehran, Iran

## Abstract

**Background:**

Celiac disease (CD) is an autoimmune disease, and its prevalence reported variously in different studies. The goal of this study is to evaluate the pooled prevalence of CD in subjects with MS.

**Methods:**

PubMed, Scopus, EMBASE, Web of Science, and Google Scholar along with gray literature were systematically searched. The search included all relevant studies which were published up to October 2022. Two researchers independently searched all databases and also references of included studies.

**Results:**

We found 8211 articles by literature search, and after deleting duplicates, 5594 remained. Fifteen articles remained for meta-analysis. Totally, 31418 patients were evaluated, and the total number of possible/confirmed cases was 124. Studies were published between 2004 and 2020, and the most published studies were from Italy. Five studies provided information regarding controls. The total number of controls was 22394, of whom 22 had CD. Mean age ranged from 35 to 55 years. The pooled prevalence of CD in MS patients was 0 (*I*^2^ = 88.2%, *p* < 0.001). The pooled odds of CD in subjects with MS are 0.46 (95% CI: 0.19-1.1) (*I*^2^ = 0, *p* = 0.9).

**Conclusion:**

The pooled prevalence of this systematic review showed that CD is not prevalent in MS cases.

## 1. Introduction

Multiple sclerosis (MS) is an autoimmune disease of central nervous system (CNS) [1, 2], affecting youth all over the world. The exact etiology of the disease is unknown, but multiple putative etiologic factors have been considered to play a role in development of MS [3].

Accompanying with a wide range of autoimmune diseases, such as hypothyroidism, inflammatory bowel disease, rheumatoid arthritis, and diabetes, could highlight common genetic or environmental exposures between MS and other autoimmune diseases [3, 4]. Epidemiological studies showed an increased susceptibility for developing another autoimmune diseases in subjects with a single autoimmune disease [5–8].

Celiac disease (CD) is an autoimmune gluten-sensitive enteropathy, which results in small intestinal lesions and malabsorption in affected cases [9]. The pathogenesis of CD is based on genetic factors and mucosal immune response [10]. Almost all affected patients with CD have HLA DR3-DQ2 and/or the DR4-DQ8 [11–13]. These HLA class II haplotypes show strong association with MS [14, 15].

On the other hand, CD is associated with neurological manifestations and diseases such as ataxia, epilepsy, neuropathy, and multiple sclerosis (MS) [16].

In some previous studies, the increased levels of anti-gliadin and gluten antibodies were detected in MS cases while another study failed to confirm this finding [9, 17, 18].

As there is no systematic review and meta-analysis regarding the prevalence of CD in MS cases, we designed this study to evaluate the prevalence of CD in MS cases.

## 2. Methods

### 2.1. Search Strategy

PubMed, Scopus, EMBASE, Web of Science, and Google Scholar along with gray literature were systematically searched. The search included all relevant studies which were published up to October 2022.

Two researchers independently searched all databases and also references of included studies.

### 2.2. The Syntax Which Was Used in MeSH Is as Follows

((Sclerosis AND multiple) OR (sclerosis AND disseminated) OR “disseminated sclerosis” OR “multiple sclerosis” OR “acute fulminating”) AND (“Celiac Disease” OR (Disease AND Celiac) OR “Gluten Enteropathy” OR (Enteropathies AND Gluten) OR (Enteropathy AND Gluten) OR “Gluten Enteropathies” OR “Gluten-Sensitive Enteropathy” OR (Enteropathies AND Gluten-Sensitive) OR (Enteropathy AND Gluten-Sensitive) OR “Gluten Sensitive Enteropathy” OR “Gluten-Sensitive Enteropathies” OR (Sprue AND Celiac) OR (Sprue AND Nontropical) OR “Nontropical Sprue” OR “Celiac Sprue” OR Sprue).

Inclusion criteria were cross-sectional studies/case, articles which had been published in the English language.

We included studies only studies in which the diagnostic criteria were biopsy of duodenum.

Exclusion criteria are letter to editors, case reports, and RCT studies.

### 2.3. Data Extraction

Two independent researchers extracted data. In the case of discrepancies, they asked another researcher. Each one entered data in an Excel sheet and data regarding the first author, country of origin, number of enrolled patients, number of CD cases, mean age, male and female numbers, mean EDSS, mean duration of the disease, number of controls, and number of CD in controls were extracted.

### 2.4. Risk of Bias Assessment

We evaluated the risk of potential bias by the Newcastle-Ottawa Quality Assessment Scale (adapted for cross-sectional studies, cohort, and case control studies) [19–21].

### 2.5. Statistical Analysis

We used STATA (version 14.0; StataCorp LP, College Station, TX, USA) for data analysis. To determine heterogeneity, inconsistency (*I*^2^) was calculated. As the *I*^2^ was more than 50%, we used random effects for pooling the data. We reported pooled prevalence with 95% CI.

## 3. Results

**Figure 1:**
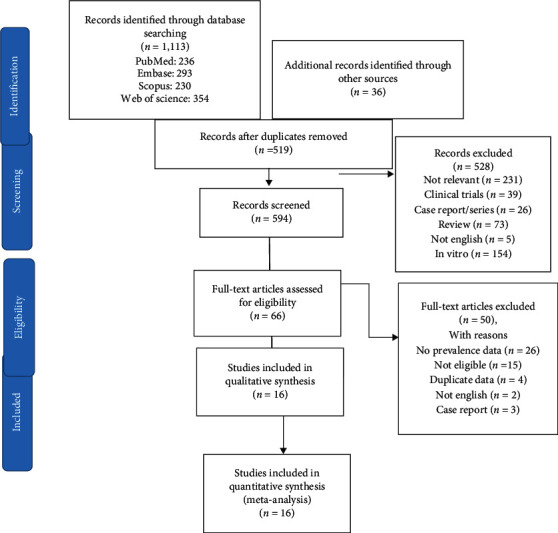
PRISMA flow diagram: the PRISMA diagram explains the intricacies of the search of systematic review and selection procedures.

We found 1113 articles by literature search, and after deleting duplicates, 519 remained. Sixteen articles remained for meta-analysis (Figure 1).

Totally, 31418 patients were evaluated and total number of possible/confirmed cases was 124. Studies were published between 2004 and 2020, and the most published studies were form Italy. Five studies provided information regarding controls. The total number of controls was 22394, of whom 22 had CD.

Mean age ranged from 35-55 years. The quality assessment score ranged between 4 and 10 (table 1).

**Figure 2:**
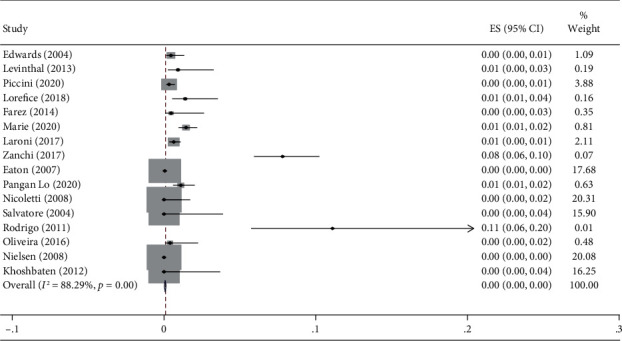
The pooled prevalence of CD in MS patients.

**Figure 3:**
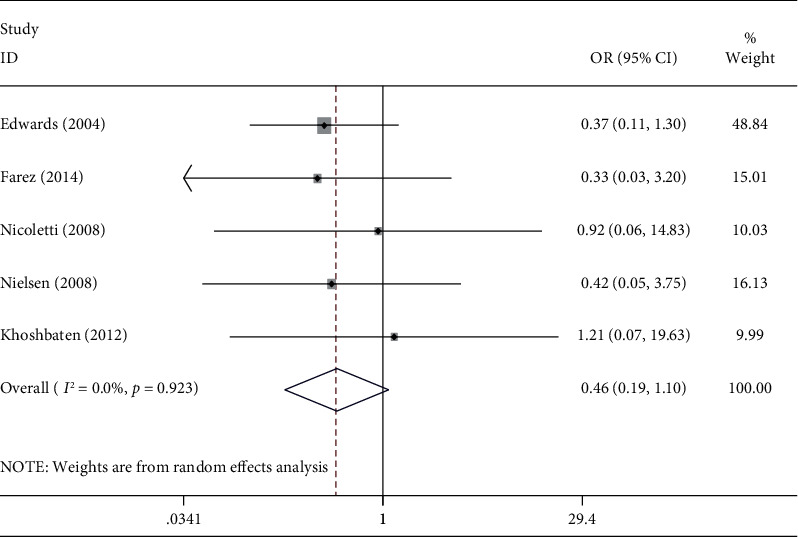
The pooled odds of CD in subjects with MS.

The pooled prevalence of CD in MS patients was 0 (*I*^2^ = 88.2%, *p* < 0.001) (Figure 2). The pooled odds of CD in subjects with MS are 0.46 (95% CI: 0.19-1.1) (*I*^2^ = 0, *p* = 0.9) (Figure 3).

## 4. Discussion

This is the first systematic review and meta-analysis evaluating the prevalence of celiac disease in MS patients.

The results show that the prevalence is near zero in MS, and the odds of CD in subjects with MS are not high.

Patients with MS suffer from a wide range of gastrointestinal manifestations such as dysphagia, constipation, and/or fecal incontinence [35–38]. Dyspeptic symptoms and pain are also common in MS cases which impair quality of life and interfere with daily activities [30].

de Oliveira et al. assessed 249 MS patients and reported CD in only one [28] which was along with findings of Nielsen et al. who evaluated gluten-sensitive enteropathy in 12403 MS cases and found it in only one (RR = 0.6, 95% CI: 0.1-4.6) [32].

Rodrigo et al. included 72 MS cases and 123 healthy controls and found the antibodies (IgA-anti-transglutaminase-2) in 10% of MS cases and 2.4% (*p* < 0.05) (OR = 5.3) while HLA-DQ2 markers did not significantly differ between patients and healthy subjects [31]. In their study, 32% of first degree had CD.

In Germany in 2001, 75 children with CD were evaluated by electroencephalogram, computed tomography (CT scan), and magnetic resonance imaging (MRI) of the brain and reported white matter lesions in 15 cases [39].

CD has a clear etiology which is autoimmunity and is the consequence of gluten intolerance. Genetics play an important role [31] and mostly occurs at adolescence [40]. The relationship between MS and CD is considered in some studies while the pathogenesis of both diseases' T-cells plays an important role, and Matheson found that patients with MS benefit from gluten free diet [41]. Shor et al. and Reichelt and Jensen reported the decrease in number of demyelinating lesions in MS cases who were treated with gluten free diet [42, 43]. They also share common HLAs [14, 15]. The prevalence of CD in different general populations is estimated between 0.2% and 0.7% [44–47].

It has been shown that CD is related with other neurological diseases such as peripheral neuropathies, seizure, ataxia, and cognitive impairment. One suggestion is that antibodies to gliadin or a peptide sequence of gliadin are neurotoxic and precede neurological manifestation in CD [48].

This systematic review had some limitations. There were studies that used serologic evaluation for CD diagnosis which were excluded. There were no reports from some countries. The control groups were different; as in some studies, the control group was healthy subjects, and in others, the control group was patients with other diseases except MS. Larger multicentric studies from lots of countries are reported.

## 5. Conclusion

The pooled prevalence of this systematic review showed that CD is not prevalent in MS cases.

## Figures and Tables

**Table 1:** Data extracted from included studies.

Author	Continent	Region	Year	Study design	Total MS cases	Female cases	Male cases	Age mean (SD)	MS stage	EDSS mean (SD) or median (range)	Total celiac case	Total control	Female control	Male control	Total celiac control	Quality assessment^∗^
Lo et al. [22]	Australia	Australia	2021	Cohort	1518	1204	309	55.7 (11.2)	NR	NR	15	NA	NA	NA	NA	7/9
Lo et al. [23]	Australia	Australia	2021	Cross-sectional	902	709	193	55.8 (11.4)	NR	NR	10	NA	NA	NA	NA	7/10
Piccini et al. [24]	Europe	Italy	2020	Cohort	2050	1579	471	28.8 (10.8)	RRMS: 1251	NR	9	NA	NA	NA	NA	7/9
Lorefice et al. [25]	Europe	Italy	2018	Cross-sectional	286	205	81	42.4 (10.6)	NR	2.7 (1.8)	4	NA	NA	NA	NA	7/10
Laroni et al. [26]	Europe	Italy	2017	Cohort	1877	1218	659	35.3 (11.3)	NR	2.1 (1.1)	12	NA	NA	NA	NA	7/9
Zanchi et al. [27]	Europe	Italy	2017	Cross-sectional	601	403	198	43.9	RRMS: 487 SPMS: 103 PRMS: 11	2 (1.0-9.0)	47	NA	NA	NA	NA	8/10
de Oliveira et al. [28]	Latin America	Brazil	2016	Cross-sectional	249	176	73	NR	NR	NR	1	NA	NA	NA	NA	8/10
Farez et al. [29]	Latin America	Argentina	2014	Case control	211	163	48	40.4 (10)	NR	1 (0-8.5)	1	211	163	48	3	7/10
Levinthal et al. [30]	America	USA	2013	Cross-sectional	218	170	48	47.6 (1.0)	RRMS: 154 SPMS: 24 PRMS: 9 Undefined: 32	NR	2	NA	NA	NA	NA	7/10
Khoshbaten	Asia	Iran	2012	Cross-sectional	100	68	32	33.1 (8.8)	RRMS: 78 SPMS: 14	3.9 (1.9)	0	121	75	46	0	6/10
Rodrigo et al. [31]	Europe	Spain	2011	Cross-sectional	72	60	12	43 (10)	RRMS: 72	1.7 (1.1)	8	Check				7/10
Nicoletti et al. [16]	Europe	Italy	2008	Case control	217	130	87	40.2 (10.2)	RRMS: 193 SPMS: 21 PRMS: 3	NR	0	200	123	77	1	8/10
Nielsen et al. [32]	Europe	Denmark	2008	Cohort	12403	NR	NR	NR	NR	NR	1	20798	NR	NR	4	
Eaton et al. [33]	Europe	Denmark	2007	Cohort	9961	NR	NR	NR	NR	NR	6	NA	NA	NA	NA	
Edwards and Constantinescu [34]	Europe	UK	2004	Case control	658	454	204	45	NR	NR	3	1064	NR	NR	13	
Salvatore et al. [17]	Europe	Italy	2004	Cross-sectional	95	NR	NR	41.3 (21-63)	RRMS: 76 SPMS: 16 PRMS: 3	NR	0	NA	NA	NA	NA	6/10
